# Resistin Enhances Inflammatory Cytokine Production in Coronary Artery Tissues by Activating the NF-*κ*B Signaling

**DOI:** 10.1155/2016/3296437

**Published:** 2016-10-09

**Authors:** Fang Gao, Feifei Si, Siqi Feng, Qijian Yi, Ruixi Liu

**Affiliations:** ^1^Department of Cardiovascular Medicine, Children's Hospital of Chongqing Medical University, CSTC2009CA5002, Yuzhong District, Chongqing 400014, China; ^2^Ministry of Education Key Laboratory of Child Development and Disorders, CSTC2009CA5002, Yuzhong District, Chongqing 400014, China; ^3^Chongqing Key Laboratory of Pediatrics, CSTC2009CA5002, Yuzhong District, Chongqing 400014, China

## Abstract

*Purpose.* Kawasaki disease (KD) is a systemic vasculitis and is a leading cause of coronary artery lesions (CALs) in childhood. Our previous study has shown higher levels of serum Resistin in KD patients with coronary aneurysm. This study aimed at examining the association of Resistin with inflammatory cytokine expression in mouse model of coronary arteritis and determining the potential mechanisms.* Methods.* C57BL/6 mice were injected with* Lactobacillus* cell wall extract (LCWE) to induce coronary arteritis. The relative levels of Resistin, TNF-*α*, IL-1*β*, and MMP-9 expression and inflammatory infiltrates in the coronary arteries were determined longitudinally by quantitative RT-PCR, ELISA, and histology. The effect of TLR4 and NF-*κ*B activation on Resistin-induced TNF-*α* and IL-1*β* expression in human coronary artery endothelium cells (HCAECs) was examined by quantitative RT-PCR.* Results.* Higher levels of Resistin, TNF-*α*, IL-1*β*, and MMP-9 expression were associated with the degrees of inflammatory infiltrates in the coronary artery walls of the LCWE-injected mice. Resistin enhanced TNF-*α* and IL-1*β* expression in HCAECs at 18 or 24 hours after stimulation. Pretreatment with anti-TLR4 attenuated Resistin-enhanced IL-1*β*, but not TNF-*α*, expression and pretreatment with parthenolide or QNZ demolished Resistin-enhanced TNF-*α* expression in HACECs. Pretreatment with parthenolide, but not QNZ, blocked Resistin-enhanced IL-1*β* expression in HCAECs.* Conclusion.* Resistin may enhance inflammation by cross-talking with TLR4/NF-*κ*B signaling during the development of coronary arteritis in mice.

## 1. Introduction

Kawasaki disease (KD) is an acute and systemic autoimmune vasculitis that primarily affects medium-sized blood vessels in young children. KD has a particularly high prevalence in Asian [1] and is the most common cause of acute vasculitis and acquired cardiac disease in developing countries [2]. Patients with KD usually display high fever, skin rash, oral mucosal redness, conjunctivitis, and joint pain and swelling [3]. During the pathogenic process, activation of vascular endothelial cells and increased serum levels of proinflammatory cytokines lead to blood vessel inflammation and injury [4, 5]. Without proper treatment, 20–25% of KD patients will develop coronary arteritis lesions (CALs) or aneurysms [6]. Although little is known about factors causing CALs in KD patients, previous studies have suggested that acute inflammation and immune system dysfunction contribute to the pathogenic process of CALs in KD patients [7]. However, the molecular mechanisms underlying the development of CALs during the process of KD have not been fully elucidated.

Resistin is an adipokine and can increase plasma levels of low density lipoproteins, associated with the development of obesity and cardiovascular diseases. Resistin can be secreted by inflammatory cells [8, 9]. Furthermore, Resistin can promote the production of inflammatory cytokines, such as interleukin-1 (IL-1), IL-6, IL-12, and tumor necrosis factor-*α* (TNF-*α*) by activating the NF-*κ*B signaling in human articular chondrocytes [10, 11]. In addition, Resistin can induce the migration and proliferation of vascular smooth muscle cells [12] and has been considered as a predictive factor for atherosclerosis progression [13] and vascular occlusion in inflammatory diseases [14]. Our previous study has shown significantly higher levels of serum Resistin in KD patients with coronary aneurysm, relative to those only with coronary artery dilatation and without CALs [15]. However, it is unclear how Resistin regulates the development of CALs during the KD process.

Toll-like receptor 4 (TLR4) is a receptor of lipopolysaccharide and others and is expressed by many types of cells [16]. Engagement of TLR4 by its ligand can activate the NF-*κ*B signaling and induce TNF-*α*, IL-1*β*, IL-6, and MMP-9 expression [17]. A recent study has shown that Resistin binds to its receptor of adenylyl cyclase-associated protein 1 (CAP1) to increase cyclic AMP concentration and protein kinase 1 activity, leading to the NF-*κ*B-related proinflammatory cytokine expression in monocytes [18]. However, it is unclear whether the Resistin-CAP1-related signaling regulates TLR4/NF-*κ*B-associated inflammatory cytokine expression during the process of KD-related CALs and in human coronary artery endothelial cells (HCAECs).

In the present study, we examined the dynamic changes in Resistin, TNF-*α*, IL-1*β*, and MMP-9 expression during the process of KD-related CALs in a mouse model of* Lactobacillus* cell wall extract- (LCWE-) induced coronary arteritis. Furthermore, we tested the effect of TLR4/NF-*κ*B signaling on Resistin-induced TNF-*α* and IL-1*β* expression in HCAECs. Our results indicated higher levels of Resistin, TNF-*α*, IL-1*β*, and MMP-9 expression in the CALs. Resistin induced TNF-*α* and IL-1*β* expression, which was regulated by the TLR4 and NF-*κ*B signaling in HCAECs. Therefore, Resistin may be important for the development of KD-related CALs and a potential therapeutic target for CALs in KD.

## 2. Materials and Methods

### 2.1. LCWE

LCWE was prepared as previously described [19]. The concentrations of the prepared LCWE in PBS were determined by measuring the rhamnose contents of the LCWE by a phenol-sulfuric acid colorimetric assay and expressed as micrograms per milliliter in PBS.

### 2.2. Mice

Wild-type C57BL/6 mice at 6–8 weeks of age were purchased from the Animal Centre of Chongqing Medical University (Chongqing, China). The mice were injected intraperitoneally with 0.5 mL of PBS alone or PBS containing 0.5 mg of LCWE to induce coronary arteritis and sacrificed at 3, 7, 14, 28, 42, and 60 days after induction [20]. The experimental protocols were approved by the Animal Care Committee of Children's Hospital of Chongqing Medical University.

### 2.3. Cardiac Histology and Immunohistochemistry

Cardiac tissues of individual mice were dissected out and fixed in 4% paraformaldehyde at room temperature for 24h, followed by dehydration, clearing, infiltration, and crystal-embedding. The coronary arteries in each sample were identified and cut into serial sections (6 *μ*m). The sections were stained with hematoxylin and eosin (H&E) and the severity of coronary arteritis was evaluated under a light microscope in a blinded manner.

The serial heart cryosections (6 *μ*m) were treated with 5% BSA (Sigma-Aldrich, USA) in PBS and stained with monoclonal rabbit anti-mouse Resistin (NOVUS Biologicals, USA) or an isotype control. After being washed, the bound antibodies were detected with biotinylated goat anti-rabbit IgG (ISGB-BIO, China) and visualized with HRP-conjugated streptavidin and DAB. Images were captured under a light microscope (Nikon C-HGFI, Japan) using NIS-Elements F 3.2 software.

### 2.4. Enzyme-Linked Immunosorbent Assay (ELISA)

Murine hearts were homogenized in lysis buffer (Ray Biotech, USA), and total proteins were extracted, according to the manufacturer's instructions. The levels of TNF-*α*, IL-1*β*, and MMP-9 were assayed using commercial ELISA kits, according to the manufacturer's instructions (Ray Biotech, USA).

### 2.5. Cells Culture and Stimulation

Human coronary artery endothelial cells, HCAECs, (ATCC, USA) were cultured in DMEM GlutaMAX (Gibco, USA) supplemented with 10% fetal bovine serum (FBS) (Gibco), penicillin, and streptomycin in a humidified condition with 5% CO_2_ at 37°C. The cells were treated in triplicate with, or without, 500 ng/mL (an optimal concentration) of recombinant human Resistin (PeproTech, UK) for varying periods (0–24 hours). In addition, the cells were pretreated with different doses of anti-TLR4, parthenolide (10 *μ*M), an inhibitor for the NF-*κ*B [16], or 40 nM QNZ [20] (Selleckchem, USA) and stimulated with 500 ng/mL of Resistin for 18 or 24 hours.

### 2.6. RNA Isolation and Quantitative Real Time RT-PCR

Total RNA from harvested cells and murine hearts was extracted using RNA isolation kit (BioTeke, China). After quantification and qualification by spectrophotometry, RNA samples were reversely transcribed into cDNA using a reverse transcriptase kit (Takara, Japan). The relative levels of target gene mRNA transcripts to control *β*-actin in individual samples were determined by quantitative real time PCR in an ABI PRISM 7000 Sequence Detection System (Applied Biosystems). Data were normalized to the control *β*-actin and analyzed by the 2^−ΔΔCT^ method.

### 2.7. Statistical Analysis

Data are expressed as mean ± SD. The difference among groups was determined by one-way ANOVA and the difference between groups was analyzed by unpaired Student's *t*-test using statistical package for social science (SPSS) statistical software version 18.0. A two-tailed *P*-value of <0.05 was considered statistically significant.

## 3. Results

### 3.1. Resistin Is Expressed in the Affected Cardiac Tissues during the Disease Process

**Figure 1:**
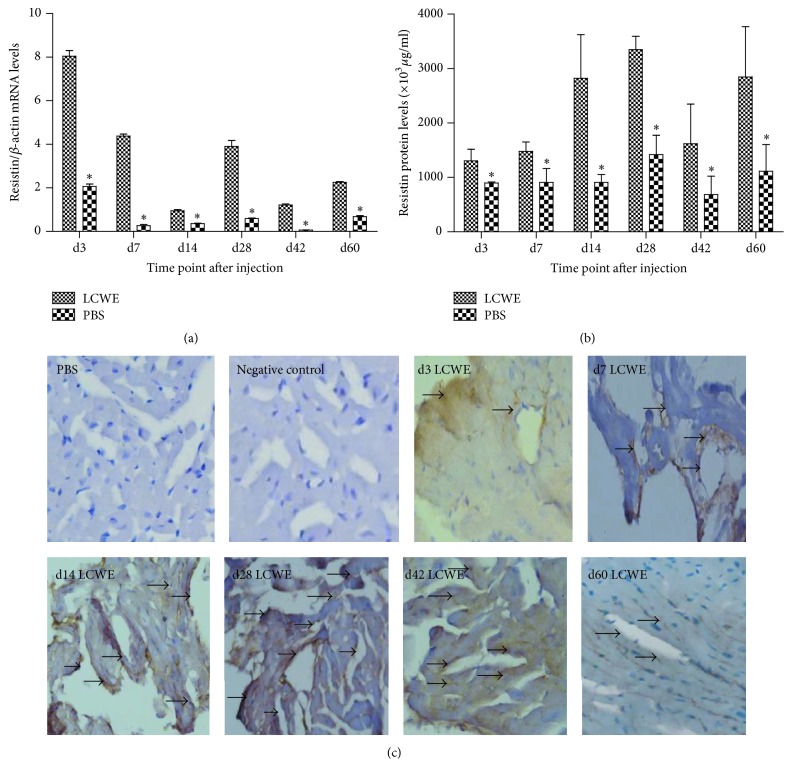
The dynamic change in Resistin expression in the coronary arteries of the LCWE-injected mice. C57BL/6 mice were injected with LCWE (0.5 mg) to induce coronary arteritis and the levels of Resistin expression in the coronary artery tissues of the mice at the indicated time points after induction were determined longitudinally by quantitative RT-PCR (a), ELISA (b), and immunohistochemistry (c). Data are representative images (magnification ×200) or expressed as the mean ± SD of each group (*n* = 3 per time point) from three separate experiments. Scale bar represents 50 *μ*m in all images. ^*∗*^ *P* < 0.05 versus the LCWE group.

To investigate the potential role of Resistin in the inflammatory process, a mouse model of coronary arteritis was induced by injection with LCWE. The levels of Resistin expression in the affected vessels of mice were determined by quantitative RT-PCR and ELISA. The relative levels of Resistin mRNA transcripts in the heart tissues of the LCME-injected mice were significantly higher than that in the PBS-treated control mice (*P* < 0.05 for all time points, Figure 1(a)). The highest levels of Resistin mRNA transcripts were detected in the hearts of mice at 3 days after induction and high levels of them were detected at 7 and 28 days after induction. Similarly, significantly higher levels of Resistin protein were detected in the hearts of mice injected with LCWE than that in the mice with PBS between 14 and 60 days after induction (*P* < 0.05, Figure 1(b)). Immunohistochemistry indicated that Resistin expression was mainly detected in the affected coronary artery wall of the LCME-injected mice, but not in the PBS-injected mice (Figure 1(c)). Hence, Resistin expression was induced in the coronary artery walls following induction of coronary arteritis in mice.

### 3.2. Resistin Expression Is Associated with Inflammation in the Coronary Artery of Mice

**Figure 2:**
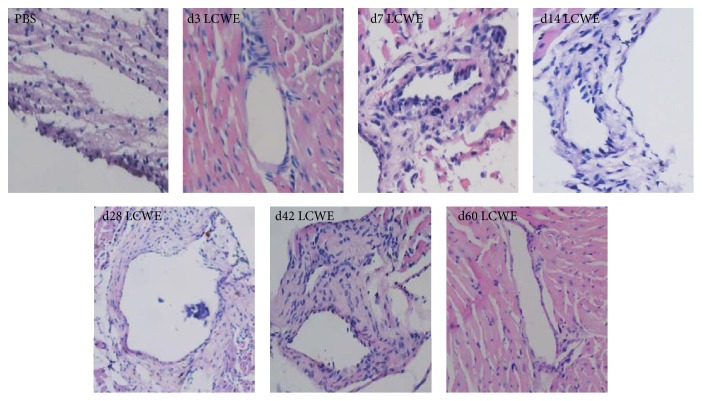
Histological examination of inflammation in the coronary artery tissues. C57BL/6 mice were injected with LCWE (0.5 mg) to induce coronary arteritis and the coronary artery tissue sections from individual groups of mice at the indicated time points after induction were stained with H&E. Data are representative images (magnification ×200) from each group (*n* = 3). Scale bar represents 50 *μ*m in all images.

Next, the inflammatory response in the coronary arteries during the disease process in mice was determined by histology. There was no detectable inflammatory infiltration in the PBS-injected mice. In contrast, 27 out of 30 mice with LCME injection developed coronary arteritis (Table 1). Inflammatory infiltrates around the coronary artery walls were detected at 3 days after induction and gradually increased during the disease process with maximal inflammatory infiltrates observed at 28 and 42 days after LCWE injection (Figure 2). Interestingly, the dynamic distribution of inflammatory infiltrates was similar to the dynamic pattern of Resistin expression in the affected coronary arteries in the LCME-injected mice throughout the observation period (Figures 1 and 2).

**Figure 3:**
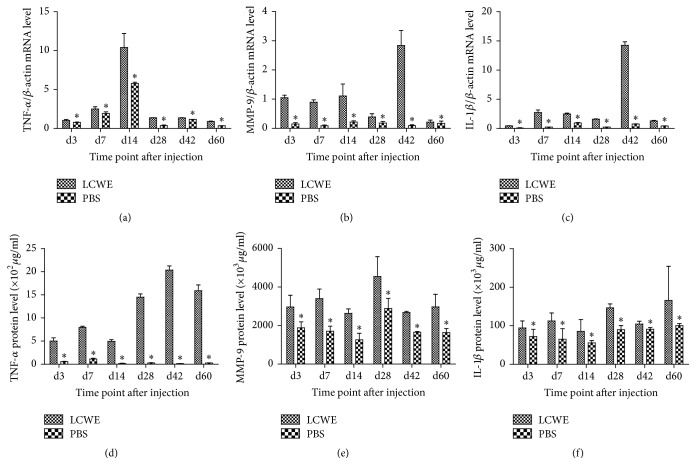
Quantitative analysis of the levels of TNF-*α*, MMP-9, and IL-1*β* expression in the heart tissues of mice. C57BL/6 mice were injected with LCWE (0.5 mg) to induce coronary arteritis and the levels of TNF-*α*, MMP-9, and IL-1*β* expression in the hearts of individual groups of mice at the indicated time points after induction were determined by quantitative RT-PCR and ELISA. Data are expressed as the mean ± SD of each group (*n* = 3 per group per time point) of mice from three separate experiments. (a–c) RT-PCR analysis of the TNF-*α*, MMP-9, and IL-1*β* mRNA transcripts. (d–f) ELISA analysis of the levels of TNF-*α* (×10^2^ *μ*g/mL), MMP-9 (×10^3^ *μ*g/mL), and IL-1*β* (×10^3^ *μ*g/mL) proteins. ^*∗*^ *P* < 0.05 versus the LCWE group.

TNF-*α*, IL-1*β*, and MMP-9 are inflammatory factors. To further understand the role of Resistin, we examined the levels of TNF-*α*, IL-1*β*, and MMP-9 expression in the hearts of different groups of mice by quantitative RT-PCR and ELISA. As shown in Figure 3, the relative levels of TNF-*α*, IL-1*β*, and MMP-9 mRNA transcripts and proteins in the hearts of mice with LCME infection were significantly higher than that in the PBS-injected controls (*P* < 0.05 for all at all time points, Figure 3). The peak levels of TNF-*α*, IL-1*β*, and MMP-9 mRNA transcripts in the hearts of mice were detected at 14, or 42 days after induction while the peak levels of TNF-*α*, IL-1*β*, and MMP-9 proteins in the hearts of mice were detected at 28–42, 28, or 60 days after induction. These suggest that the levels of Resistin expression may be associated with the severity of coronary arteritis in mice.

### 3.3. Resistin Stimulates Proinflammatory Cytokine Expression in HCAECs

**Figure 4:**
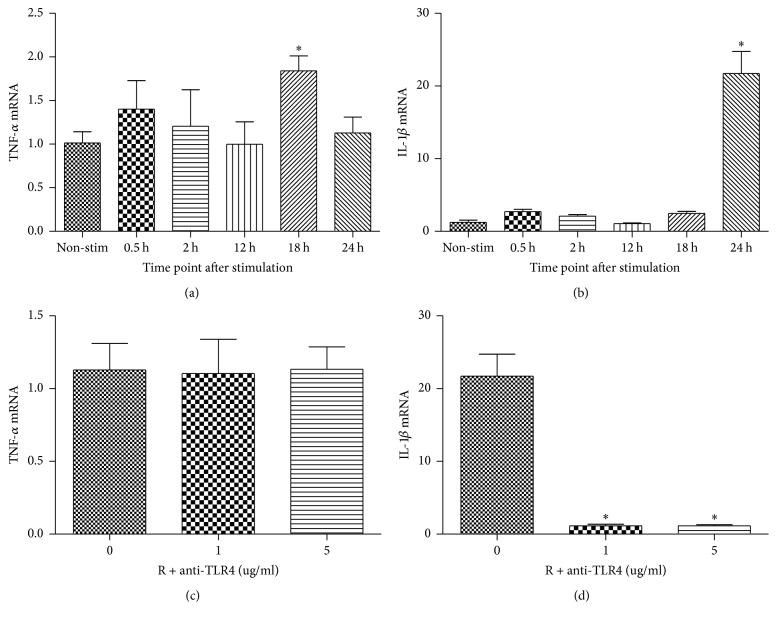
Resistin stimulates TNF-*α* and IL-1*β* production in HCAECs. HCAECs were stimulated in triplicate with Resistin (500 ng/mL) for varying periods and the relative levels of TNF-*α* and IL-1*β* mRNA transcripts were determined by quantitative RT-PCR ((a) and (b)). In addition, HCAECs were pretreated with the indicated doses of anti-TLR4 (0, 1, or 5 *μ*g/mL) and stimulated with Resistin (500 ng/mL) for 18 or 24 hours, respectively, and the relative levels of TNF-*α* and IL-1*β* mRNA transcripts were determined. Data are expressed as the mean ± SD of each group of cells from three separate experiments ((c) and (d)). ^*∗*^ *P* < 0.001 versus the control cells without stimulation. R: Resistin, anti-TLR4: TLR4 antibody, and Non-stim: nonstimulation.

To assess the functional role of Resistin in proinflammatory responses, HCAECs were treated with 500 ng/mL of Resistin for varying periods and the relative levels of TNF-*α* and IL-1*β* mRNA transcripts were determined by quantitative RT-PCR. Treatment with Resistin induced significantly higher levels of TNF-*α* and IL-1*β* mRNA transcripts in HCAECs at 18 and 24 hours after stimulation, respectively (*P* < 0.001, Figures 4(a) and 4(b)). To explore the potential role of TLR4 in Resistin-induced proinflammatory cytokine expression, HCAECs were pretreated with TLR4 antibodies (at a dose of 0, 1, and 5 *μ*g/mL), stimulated with Resistin (500 ng/mL) for 18 or 24 hours, and assessed for the expression of TNF-*α* and IL-1*β* by quantitative RT-PCR. Pretreatment with anti-TLR4 did not change the Resistin-induced TNF-*α* mRNA transcripts but significantly reduced the Resistin-induced IL-1*β* mRNA transcripts in HCAECs (*P* < 0.001, Figures 4(c) and 4(d)). Hence, Resistin induces both proinflammatory TNF-*α* and IL-1*β* expressions in HCAECs, and the IL-1*β*, but not TNF-*α*, expression induced by Resistin was dependent on TLR4 in HCAECs.

### 3.4. Resistin Simulates Proinflammatory TNF-*α* and IL-1*β* Expression in HCAECs Dependent on the NF-*κ*B Activation

**Figure 5:**
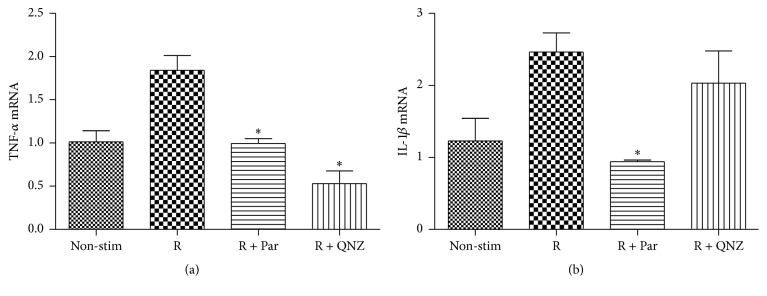
Resistin stimulates TNF-*α* and IL-1*β*, depending on the NF-*κ*B activation in HCAECs. HCAECs (1 × 10^5^/mL) were pretreated in triplicate with, or without, parthenolide (10 *μ*M) or QNZ (40 nM) and stimulated in triplicate with Resistin (500 ng/mL) for 18 or 24 hours. The relative levels of TNF-*α* and IL-1*β* mRNA transcripts were determined by quantitative RT-PCR. Data are expressed as the mean ± SD of each group of cells from three separate experiments. (a) The levels of TNF-*α* transcripts. (b) The levels of IL-1*β* transcripts. ^*∗*^ *P* < 0.05 versus the Resistin group. Non-stim: nonstimulation, R: Resistin, and Par: parthenolide.

Finally, we examined whether Resistin-induced TNF-*α* and IL-1*β* expression was dependent on the NF-*κ*B activation in HCAECs. HCAECs were pretreated with, or without, parthenolide (10 *μ*M), an inhibitor of the classical NF-*κ*B pathway or with QNZ (40 nM), an inhibitor of the nonclassical NF-*κ*B pathway, for one hour and treated with, or without, Resistin for 18 or 24 hours. The relative levels of TNF-*α* and IL-1*β* mRNA transcripts in the different groups of cells were determined by quantitative RT-PCR. Treatment with either parthenolide or QNZ alone did not change the relative levels of TNF-*α* and IL-1*β* expression in HCAECs (data not shown). Pretreatment with either parthenolide or QNZ significantly decreased the Resistin-elevated TNF-*α* mRNA transcripts in HCAECs (*P* < 0.05, Figure 5(a)). Pretreatment with parthenolide, but not with QNZ, significantly mitigated the Resistin-induced IL-1*β* mRNA transcripts in HCAECs (*P* < 0.05, Figure 5(b)). Therefore, Resistin induces proinflammatory TNF-*α* and IL-1*β* expression in HCAECs, dependent on the NF-*κ*B activation.

## 4. Discussion

Our previous study has shown high levels of serum Resistin in KD patients with coronary aneurysms [15]. In this study, we employed a mouse model of coronary arteritis to determine the dynamic changes in the levels of Resistin expression in coronary artery tissues during the pathogenic process of coronary arteritis. We found that the levels of Resistin expression increased during the pathogenic process of coronary arteritis in mice, associated with gradually increased inflammatory infiltrates in the coronary artery tissues. Furthermore, the increased levels of Resistin expression were accompanied by upregulated TNF-*α*, IL-1*β*, and MMP-9 expression in coronary artery tissues in the LCWE-injected mice. These data suggest that Resistin may be pathogenic factor to enhance inflammation, contributing to the pathogenic process of coronary arteritis in mice. Given that the levels of Resistin expression were positively associated with the levels of proinflammatory cytokines and the degrees of inflammatory infiltrates in the coronary artery tissues of mice Resistin may be a valuable biomarker for evaluating the severity of coronary arteritis.

A previous study has shown that TNF-*α* is crucial for the development of coronary aneurysms in KD patients [21]. In this study, we found higher levels of Resistin, TNF-*α*, MMP-9, and IL-1*β* in the coronary artery tissues of the LCWE-injected mice. Because Resistin can induce proinflammatory TNF-*α* and IL-1*β* expression in monocytes [16], it is possible that the high levels of Resistin may enhance TNF-*α* and IL-1*β* production during the pathogenic process of coronary arteritis in LCWE-injected mice. Subsequently, the high levels of TNF-*α* and IL-1*β* may promote the migration and adhesion of inflammatory neutrophils and macrophages to vessel endothelium [22]. These, together with high levels of MMP-9 that can break down elastin in coronary artery walls, promote the development of coronary aneurysms during the pathogenic process of KD. Actually, blockage of TNF-*α* has been shown to inhibit the development of coronary aneurysms in LCWE-injected mice [22]. Given that high levels of serum Resistin are detected in KD patients with coronary aneurysms it is possible that Resistin may enhance inflammatory cytokine production and inflammatory cell infiltration in the coronary artery walls, contributing to the development of CALs during the pathogenic process of KD in humans. Hence, Resistin may be a therapeutic target for intervention of KD.

To further understand the role of Resistin in the development of coronary artery lesions, we examined the effect of Resistin on TNF-*α* and IL-1*β* expression in HCAECs and we found that Resistin stimulated TNF-*α* and IL-1*β* expression in HCAECs. These data extended our previous observation that Resistin promoted MMP-9 expression in HCAECs [23]. Given that Resistin binds to its receptor CAP1 and the TLR4/NF-*κ*B signaling is crucial for TNF-*α* and IL-1*β* expression [21], we further investigated the effect of anti-TLR4 or inhibitors for the NF-*κ*B signaling on Resistin-stimulated TNF-*α* and IL-1*β* expression in HCAECs. We found that pretreatment with anti-TLR4 blocked the Resistin-stimulated IL-1*β*, but not TNF-*α*, expression, indicating that Resistin-induced IL-1*β* expression depends on TLR4-related signaling in HCAECs. In contrast, Resistin induced TNF-*α* expression in HCAECs, independent of TLR4-related signaling. Furthermore, we found that pretreatment with parthenolide or QNZ to inhibit the classical or nonclassical NF-*κ*B signaling completely abrogated Resistin-induced TNF-*α* expression in HCAECs. In contrast, pretreatment with parthenolide, but not QNZ, significantly attenuated the Resistin-stimulated IL-1*β* expression in HCAECs. These novel data indicated that Resistin stimulated IL-1*β* expression by activating the TLR4/classical NF-*κ*B signaling while Resistin induced TNF-*α* expression by crosstalk of the Resistin/CAP1-related signaling with the NF-*κ*B signaling, but independent of TLR4 in HCAECs. Therefore, Resistin induces TNF-*α* and IL-1*β* expression through activating different pathways in HCAECs. We are interested in further investigating the molecular mechanisms underlying the regulation of Resistin on proinflammatory cytokine production during the pathogenic process of coronary arteritis and KD.

In summary, our data indicated that high levels of Resistin, TNF-*α*, IL-1*β*, and MMP-9 expression were associated with the levels of proinflammatory cytokines and the degrees of inflammatory infiltrates in coronary artery tissues of the LCWE-injected mice. These findings suggest that Resistin may be a pathogenic factor, contributing to the development of coronary arteritis in mice, and a biomarker for evaluating the severity of coronary arteritis. Furthermore, we found that Resistin stimulated TNF-*α* and IL-1*β* expression in HCAECs by activating the NF-*κ*B signaling. Our findings may provide new insights into the pathogenesis of CALs during the process of KD.

## Figures and Tables

**Table 1:** The incidence of coronary artery lesions after LCWE injection and PBS injection.

Mouse strain	Incidence of inflammation	
	LCWE	PBS
Wild type	27/30	0/20
